# Keratoconus and Keratoectasia: Advancements in Diagnosis and Treatment

**DOI:** 10.1155/2012/526058

**Published:** 2012-02-29

**Authors:** Antonio Leccisotti, Ioannis M. Aslanides, Johnny E. Moore, Sunil Shah

**Affiliations:** ^1^School of Biomedical Sciences, University of Ulster, Cromore Road, Coleraine, BT52 1SA, UK; ^2^Siena Eye Laser, 53035 Monteriggioni, Siena, Italy; ^3^Emmetropia Mediterranean Eye Institute, Plateia Eleytherias 44, 71201, Heraklion, Crete, Greece; ^4^School of Life & Health Sciences, Aston University, Aston Triangle, Birmingham, B4 7ET, UK; ^5^Midland Eye Institute, 50 Lode Lane, Solihull, West Midlands, B91 2AW, UK; ^6^Heart of England Foundation Trust, Bordesley Green East, Birmingham, B9 5SS, UK; ^7^Birmingham & Midland Eye Centre, Sandwell and West Birmingham NHS Trust City Hospital Dudley Road, Birmingham, B187QH, UK

Keratoconus (KC) and iatrogenic keratoectasia are receiving increasing attention, due to the improvements in diagnostic modalities and the availability of therapeutic options, which now include collagen cross-linking, intrastromal implants, intraocular lenses, microwave remodeling, and anterior lamellar keratoplasty.

Limitations of surgical treatments of keratoconus are well known. Intrastromal implants, built in various shapes and now implanted more safely through femtosecond-laser-obtained stromal channels, still retain reduced predictability as for the refractive results and do not modify the structure of the diseased cornea. Anterior lamellar keratoplasty, even in its more advanced and technically difficult variant of deep anterior lamellar keratoplasty (DALK), cures the disease by the (almost) complete replacement of the ectatic stroma but, even when a regular and transparent interface is achieved, final refractive errors and higher-order aberrations may severely affect visual rehabilitation. The use of femtosecond laser in DALK to shape the donor and recipient margins has not significantly improved the picture yet.

Parasurgical treatments of KC are therefore regarded as a temporary or definitive alternative to surgical interventions. Among the newest ideas, the promising use of microwave to heat and reshape the corneal apex shares the principle with previous modalities of thermal keratoplasty, which were characterized by regression and induction of irregular astigmatism. The long-term validity of microwave reshaping is, therefore, still being investigated.

The use of collagen corneal cross-linking (CXL) with riboflavin and ultraviolet (UV) has rapidly expanded in the world and is currently regarded as the only recognized treatment to slow or arrest KC progression, obtaining in some cases a significant improvement of corneal curvature and regularity. However, as most new treatments, CXL is still far from being ideal. Riboflavin for CXL is unreasonably expensive; the treatment is long and tedious and is followed by postoperative pain and slow visual rehabilitation. Complications are not uncommon, including infections and scarring. The indications to the treatment are still debated as for age, KC stage, and corneal thickness. Alternative attempts to reduce the CXL operating time by increasing the irradiation energy or by avoiding epithelial removal have been made, but all deviations from the defined original protocol may reduce the efficacy of treatment, and therefore new treatment protocols are currently further investigated.

In this special issue, various and new aspects of CXL are examined, rehabilitation with contact lenses of KC is reviewed, and the features of posterior KC at ultrasound biomicroscopy are evaluated.

Patient selection for CXL is not completely codified, and age limits are conventionally established. For example, the Italian National Health Service limits CXL reimbursement for patients between 12 and 40 years, the lower limit being dictated by common sense and the upper limit by the presumption of spontaneous KC stabilization after 40. A. Caporossi and Mazzotta et al., leading experts of CXL, in their original study in this issue, compare KC stabilization, improvement of corneal curvature, visual acuity, and aberrations 48 months after CXL in different age groups, concluding that the highest benefits were obtained in younger eyes.

CXL procedure was originally developed to stiffen the keratoconic cornea, but its indications have been recently extended to postrefractive surgery ectasia, to infectious keratitis (due to a powerful antimicrobial action), and to corneal edema, where CXL temporarily reduces the space for fluid accumulation. These new indications of CXL, as well as its physical and chemical background, biomechanical effects, and clinical results, are thoroughly reviewed in the paper by M. Hovakimyan et al., where the real possibilities of transepithelial CXL and of the new approach combining photorefractive keratectomy (PRK) and CXL are discussed.

Several reports of infectious keratitis after CXL have recently raised the issue of CXL safety: it would appear that the risk of infection is considerably higher than after PRK. The length of the procedure or the slow epithelialization time could be the reasons for such increased infectious risk. In addition, the peculiar “demarcation” haze, regarded as a demonstration of the cross-linking effect, can sometimes turn into a significant, long-term scar. These complications and others are well reviewed in the paper by S. Dhawan et al.

Fortunately, most patients with KC will never need to undergo any surgical or parasurgical procedure. Visual rehabilitation is sometimes possible with the sole help of spectacles, but the reduction of higher-order aberrations is only possible with contact lenses. The extended wear of contact lenses and the difficult adaptation in keratoconic eyes imply a thorough knowledge of various contact lens models available: this is the subject of the article by Ozkurt et al.

The paper by B. Rejdak et al. is a case report of a rare, nonprogressive variant of KC, circumscribed posterior keratoconus. The correct diagnosis of this form of ectasia is only possible by modern three-dimensional imaging technique, and in this case ultrasound biomicroscopy and slit scanning topography were used to reveal the protrusion of the posterior corneal surface.

In this historical period we are directly witnessing the rise (and fall) of many therapeutic modalities for KC, but we can nevertheless look with optimism at the future of a complex and multiform disease, characterized by individualised treatment and prognosis. We hope that this special issue will contribute to stimulating discussion.

*Antonio Leccisotti* *Antonio Leccisotti* *Ioannis M. Aslanides* *Ioannis M. Aslanides* *Johnny E. Moore* *Johnny E. Moore* *Sunil Shah* *Sunil Shah*

